# Ipriflavone Inhibits Porcine Reproductive and Respiratory Syndrome Virus Infection via RIG-I/IRF3-Mediated Interferon Signaling

**DOI:** 10.3390/ani15192840

**Published:** 2025-09-29

**Authors:** Yafei Chang, Zhaopeng Li, Kanglei Pei, Mengqi Wang, Xiaobo Chang

**Affiliations:** 1 College of Animal Science and Veterinary Medicine, Henan Institute of Science and Technology, Xinxiang 453003, China; changyafei1990@163.com (Y.C.); 18317726729@163.com (Z.L.); peikangl24@163.com (K.P.); w15137107678@163.com (M.W.); 2 Postdoctoral Innovation Practice Base, College of Animal Science and Veterinary Medicine, Henan Institute of Science and Technology, Xinxiang 453003, China

**Keywords:** PRRSV, ipriflavone, antiviral response, RIG-I, IRF3

## Abstract

Porcine reproductive and respiratory syndrome virus (PRRSV) remains one of the most important pathogens, resulting in huge economic losses to the global pig industry. Ipriflavone is an isoflavone derivative involved in various biological processes, showing anti-inflammatory, anti-apoptotic, antioxidant, and neuroprotective effects. However, the role of ipriflavone in antiviral immune response to PRRSV is unknown. In this study, we discovered that ipriflavone could significantly inhibit PRRSV replication. Moreover, ipriflavone inhibited PRRSV replication regardless of whether ipriflavone was added pre-, co-, or post-PRRSV infection, and ipriflavone mainly inhibited virus replication and assembly stages. Importantly, ipriflavone had the capacity to upregulate the expression levels of IFN-β and ISG56. Additionally, ipriflavone promoted the expression of RIG-I and MAVS, and induced phosphorylation of IRF3 and STAT1, while reducing PRRSV replication. Collectively, ipriflavone could enhance the RIG-I/IRF3 signaling pathway, thereby inhibiting PRRSV replication. These findings will provide an important theoretical basis for the development of therapeutic agents against PRRSV infection.

## Simple Summary

PRRSV remains one of the most significant pathogens that pose a serious threat to the pig farming industry. Ipriflavone is an isoflavone derivative available in various biological processes. In this study, we demonstrated that ipriflavone has anti-PRRSV activity and mainly disturbed PRRSV replication and assembly stages. In addition, ipriflavone could inhibit PRRSV replication via enhancing the RIG-I/IRF3-mediated I-IFN signaling pathway. These findings will provide insights into a potential therapy for PRRSV control.

## 1. Introduction

Porcine reproductive and respiratory syndrome virus (PRRSV), the causative pathogen of porcine reproductive and respiratory syndrome (PRRS), is an enveloped virus with a single-stranded and positive-sense RNA genome containing at least 10 open reading frames (ORFs) [1,2,3]. The N protein, encoded by ORF7, plays a pivotal role in the process of virus replication [4]. PRRSV belongs to the *Nidovirales* order, and can be categorized into two genotypes, namely PRRSV-1 and PRRSV-2 [5,6]. PRRSV-1 is further divided into 4 lineages, whereas PRRSV-2 is split into 11 lineages [7,8]. As a result, high genetic diversity is a key characteristic of PRRSV. Currently, vaccination is the main method for preventing PRRS [9,10]. Nevertheless, the existing commercially available vaccines cannot completely protect pigs from PRRSV infection due to the genetic diversity and the impaired immune response induced by PRRSV [11]. Thus, there is an urgent need to develop new effective therapies to combat PRRSV.

Type I interferons (I-IFNs) and interferon-stimulated genes (ISGs) play pivotal roles in the antiviral immune response [12,13,14]. Viral nucleic acids, identified as pathogen-associated molecular patterns (PAMPs), are specifically recognized by pattern-recognition receptors (PRRs) [15]. Retinoic acid-inducible gene I (RIG-I)-like receptors (RLRs), acting as RNA sensors, could recruit the mitochondrial antiviral signaling (MAVS), which leads to the activation of interferon regulatory factor 3 (IRF3), and the production of I-IFNs [16,17,18]. Once released, these IFNs bind to interferon receptors (IFNARs) and initiate the JAK/STAT signaling cascade [19]. This cascade subsequently triggers the expression of ISGs, ultimately contributing to the inhibition of the viral replication [20]. For example, IFI16, an interferon-gamma-inducible protein, can enhance the production of IFN-β during PRRSV infection, thereby inhibiting PRRSV replication [21]. In addition, CH25H can be induced by IFN-α and PRRSV, and it exerts a significant inhibitory effect on PRRSV infection by preventing virus entry [22].

Ipriflavone, a phytoestrogen derived from natural isoflavones, is reported to possess multiple bioactive effects, such as anti-inflammatory, anti-apoptotic, antioxidant, antimutagenic, and neuroprotective [23,24,25]. It has been reported that ipriflavone could antagonize the LPS-induced activation of protein levels of NLRP3, ASC, and Caspase-1. Moreover, it can inhibit the nuclear translocation of NF-κB through antagonizing glucocorticoid receptor (GR). These findings collectively suggest that ipriflavone exerts an inhibitory effect on the NF-κB/NLRP3/ASC/Caspase-1 signaling pathway [26]. In addition, Ipriflavone can provide potential neuroprotective effect against LPS-induced neuroinflammation in rats through its anti-inflammatory and antioxidant activities. Furthermore, ipriflavone has been demonstrated to effectively alleviate the H_2_O_2_-induced cell death and reduce H_2_O_2_-induced elevations of both reactive oxygen species and apoptosis rate [27]. However, the roles of ipriflavone in the innate immunity and PRRSV replication remain unknown.

In this study, we found that ipriflavone exhibited significant anti-PRRSV activity and could interfere with virus replication and assembly. Additionally, ipriflavone increased the expression of IFN-β and ISG56. Moreover, ipriflavone was involved in RIG-I/IRF3 activation. Overall, this study elucidated the underlying mechanism by which ipriflavone inhibits PRRSV replication through regulating the RIG-I/IRF3-mediated IFN-β signaling pathway, and our findings will provide insights into a potential therapy for PRRSV control.

## 2. Materials and Methods

### 2.1. Cells and Virus

Marc-145 cells (African green monkey kidney cells, cells permissive to PRRSV) were maintained in Dulbecco’s modified Eagle’s medium (DMEM, Solarbio, Beijing, China) supplemented with 10% fetal bovine serum (FBS, Gibco, Grand Island, NY, USA) and 1% penicillin (100 U/mL)-streptomycin (100 µg/mL) (Solarbio, Beijing, China). Porcine alveolar macrophages (PAMs) were cultured in RPMI 1640 medium (Solarbio, Beijing, China) supplemented with 10% FBS and 1% penicillin-streptomycin. Marc-145 cells and PAMs were cultured in a humidified incubator with 5% CO_2_ at 37 °C. The PRRSV strain BJ-4 (GenBank accession no. AF331831) was used for all experiments.

### 2.2. Antibodies and Reagents

The anti-PRRSV N protein antibody (GTX129270) was purchased from GeneTex (Alton PkwyIrvine, CA, USA). The p-IRF3 antibody (29528-1-AP), IRF3 antibody (11312-1-AP), p-STAT1 antibody (28979-1-AP), STAT1 antibody (66545-1-Ig), RIG-I antibody (20566-1-AP), MAVS antibody (14341-1-AP), Histone H3 antibody (17168-1-AP), GAPDH antibody(60004-1-Ig), Beta Actin antibody (66009-1-Ig), HRP-conjugated goat anti-rabbit IgG (SA00001-2) and HRP-conjugated goat anti-mouse IgG (SA00001-1) were purchased from Proteintech (Wuhan, China). Goat anti rabbit IgG FITC antibody (F0382-.5ML) was purchased from Sigma-Aldrich (St. Louis, MO, USA). Ipriflavone was purchased from Solarbio (Beijing, China). Triton X-100, 4% paraformaldehyde and DAPI were purchased from Beyotime (Shanghai, China).

### 2.3. Cytotoxicity Assay

TransDetect^®^ cell counting kit (CCK) (TransGen Biotech, Beijing, China) was used to assess the cytotoxicity of ipriflavone toward Marc-145 cells. Briefly, the cells were incubated with ipriflavone for 48 h at 37 °C, then 100 μL of 10% CCK solution was added in accordance with the manufacturer’s protocol. After 1 h of incubation, absorbance was measured at 450 nm using a microplate reader.

### 2.4. Quantitative Real-Time PCR Analysis

The Cell/Tissue Total RNA Isolation Kit (Vazyme, Nanjing, China) was used for extracting the total RNA. Subsequently, the RNA was reverse transcribed using HiScript^®^ III All-in-one RT SuperMix (Vazyme, Nanjing, China). Finally, the cDNA was amplified using ChamQ Universal SYBR qPCR Master Mix (Vazyme, Nanjing, China). The relative mRNA levels were normalized to the level of GAPDH and calculated using the 2^−ΔΔCT^ method. The primers used for qPCR are listed in Table 1.

### 2.5. Western Blot

The cells were lysed on ice for 20 min using 100 μL RIPA lysis buffer (Sigma-Aldrich, St. Louis, MO, USA), which was supplemented with protease inhibitor cocktail and phosphatase inhibitor cocktail (Roche, Basel, Switzerland). Then, the quantified protein lysates were subjected to 12% SDS-PAGE and transferred to a PVDF membrane (Millipore, Darmstadt, Germany). Subsequently, the membranes were blocked with 5% skim milk (BD Biosciences, Franklin Lakes, NJ, USA) in PBST buffer for 2–3 h at room temperature, and incubated with indicated primary antibody overnight at 4 °C, followed by the incubation with appropriate HRP-conjugated goat anti-rabbit IgG or goat anti-mouse IgG for 1 h at room temperature. After washing with PBST, the distinct protein bands were detected using the ECL Western blot substrate (Thermo Fisher Scientific, Waltham, MA, USA).

### 2.6. Immunofluorescence Assay (IFA)

Marc-145 cells that had been treated with ipriflavone and PRRSV were fixed with paraformaldehyde (PFA, Beyotime, Shanghai, China) for 15 min. The cells were subsequently permeabilized with 0.3% Triton X-100 (Beyotime, Shanghai, China) for 10 min and blocked with 5% skim milk in PBST for 30 min at room temperature. Then the primary antibody (anti-PRRSV N Ab) and goat anti rabbit IgG FITC-conjugated secondary antibody were successively added. Finally, the cells were stained with DAPI (Beyotime, Shanghai, China) and the immunofluorescence was observed using a Zeiss inverted fluorescence microscope.

### 2.7. Time-of-Addition Assay

Marc-145 cells were seeded in 6-well plates at 37 °C, following the mock treatment (untreated: no ipriflavone was added), post-treatment (ipriflavone was added 2 h after PRRSV infection), co-treatment (ipriflavone and PRRSV were added together), or pre-treatment (ipriflavone was added 2 h before PRRSV infection). After washing with PBS, the samples were collected at 24 hpi and subjected to indicated assays.

### 2.8. Viral Binding, Entry, Replication, Assembly, and Release Assays

Virus binding assay. Marc-145 cells were pre-cooled at 4 °C for 2 h, and the media was replaced with DMEM containing 2% FBS, then treated with PRRSV at a multiplicity of infection (MOI) of 0.5 or 5 and ipriflavone (20 μg/mL) at 4 °C for another 2 h. Subsequently, the cells were washed with ice-cold PBS three times, and samples were collected and analyzed by qPCR. 

Virus entry assay. Marc-145 cells were cultured at 4 °C for 2 h before PRRSV infection (MOI = 5) for 2 h at 4 °C. After washing, the cells were incubated with ipriflavone or DMSO at 37 °C for 2 h. Then the samples were harvested and quantified by qPCR.

Virus replication assay. Marc-145 cells were infected with PRRSV (MOI = 0.1) at 37 °C for 6 h. Subsequently, the cells were washed three times with PBS and then treated with ipriflavone or DMSO at 37 °C. At 24 h post-infection (hpi), the samples were collected and analyzed by qPCR.

Virus assembly assay. Marc-145 cells were incubated with a mixture of PRRSV and ipriflavone at 37 °C for 2 h. The cells were washed with PBS three times and subsequently treated with ipriflavone for another 22 h. At 24 hpi, the cells and supernatants were collected for the measurement of viral titers and viral RNA.

Virus release assay. Marc-145 cells were infected with PRRSV (MOI = 0.1), and at 24 hpi, the cells were washed three times and then treated with ipriflavone or DMSO for 10, 30, and 60 min at 37 °C. Next, the cell supernatants were collected for the measurement of viral titers.

### 2.9. Virus Titration

Virus titers were measured based on a previous report [28]. Briefly, Marc-145 cells grown in 96-well plates were infected with 10-fold serial dilutions of virus samples. After incubation for 2 h at 37 °C, the culture medium was gently replaced with fresh DMEM containing 2% FBS. Subsequently, the cytopathic effect (CPE) was observed at 3–5 days post-infection (dpi) and the TCID_50_ was determined by the Reed–Muench method.

### 2.10. Statistical Analysis

All statistical analyses were conducted utilizing GraphPad Prism 9.0 software. The data are expressed as the mean and standard deviation (SD). For statistical comparison, a Student *t*-test was used. Moreover, in the figures, asterisks are used to denote statistically significant differences, and a *p*-value of <0.05 was considered statistically significant.

## 3. Results

### 3.1. Ipriflavone Inhibits PRRSV Replication In Vitro

**Figure 1:**
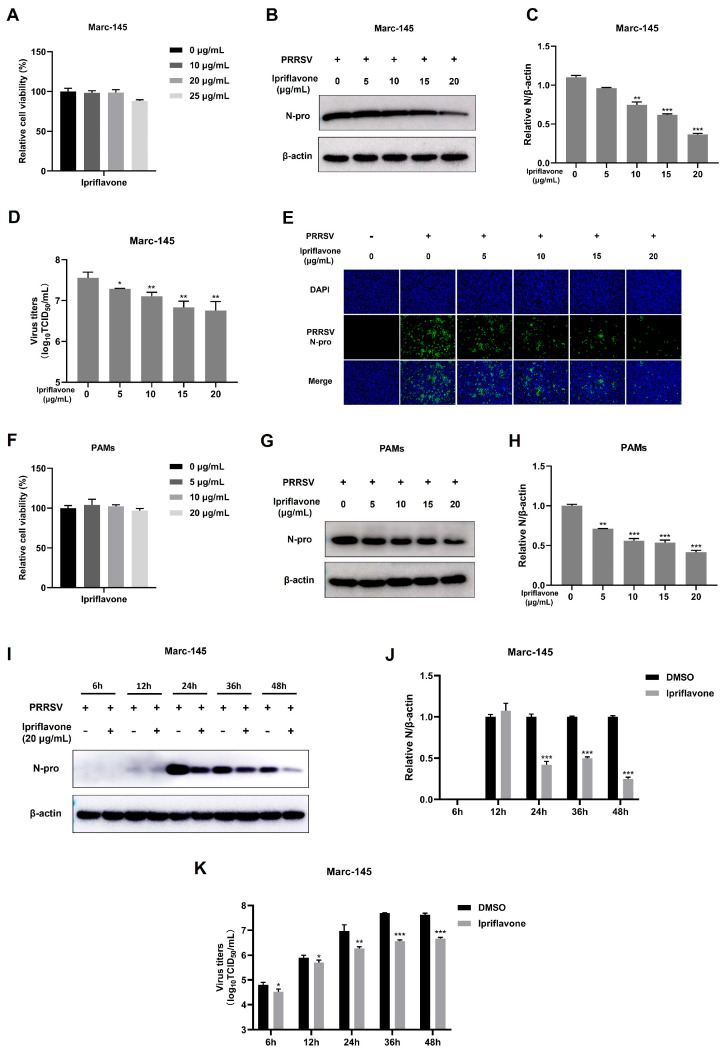
Ipriflavone significantly inhibits PRRSV replication. (**A**) After 48 h of ipriflavone treatment, the cell viability was determined by CCK-8 assay. (**B**–**E**) Marc-145 cells were infected with PRRSV (MOI = 0.1) in the presence of ipriflavone (0, 5, 10, 15, 20 μg/mL) for 24 h. (**B**) The expression levels of PRRSV N protein were detected by Western blot. (**C**) The relative expression levels of N protein were quantified using Image J 1.53e software. (**D**) The virus titers were analyzed by TCID_50_. (**E**) Immunofluorescence analysis of PRRSV N protein; Scale bar = 100 μm. (**F**) Viability of PAMs treated with the indicated concentrations of ipriflavone. (**G**,**H**) PAMs were infected with PRRSV (MOI = 1) in the presence of ipriflavone (0, 5, 10, 15, 20 μg/mL) for 24 h. (**G**) The expression levels of PRRSV N protein were detected by Western blot. (**H**) The relative expression levels of N protein were quantified using Image J software. (**I**–**K**) Marc-145 cells were infected with PRRSV (MOI = 0.1) in the absence or presence of ipriflavone (20 μg/mL). At 6, 12, 24, 36, and 48 hpi, the cells were collected. (**I**) The expression levels of PRRSV N protein were detected by Western blot. (**J**) The relative expression levels of N protein were quantified using Image J software. (**K**) The virus titers were analyzed by TCID_50_. The asterisks in the figures indicate significant differences (* *p* < 0.05, ** *p* < 0.01, *** *p* < 0.001).

To determine whether ipriflavone has an anti-PRRSV effect in vitro, we examined the cytotoxicity of ipriflavone toward Marc-145 cells using the CCK8 assay. The results showed that the cell viability of Marc-145 cells did not show a significant difference within the concentration range of 0–20 μg/mL (Figure 1A). Next, the anti-PRRSV effect of ipriflavone was evaluated by detecting the expression of PRRSV N protein at 24 h post-infection. As shown in Figure 1B,C, ipriflavone (10, 15, and 20 μg/mL) could reduce the abundance of PRRSV N protein and showed the strongest inhibitory effect at a concentration of 20 μg/mL compared to the control group. Furthermore, the results of TCID_50_ also indicated that ipriflavone significantly inhibited PRRSV infection (Figure 1D), which was consistent with the IFA results (Figure 1E).

As we know, PAMs are the main target for PRRSV infection in pigs. Therefore, the anti-PRRSV activity of ipriflavone was further explored in PAMs. Cell viability analysis showed that ipriflavone exerted little cytotoxicity on PAMs at various concentrations (Figure 1F). Western blot demonstrated that ipriflavone significantly decreased the expression of PRRSV N protein in PAMs (Figure 1G,H).

In addition, we investigated the effects of ipriflavone on PRRSV at different time points. Western blot confirmed that the expression of the PRRSV N protein was decreased at 24, 36, and 48 hpi compared to the cells untreated with ipriflavone (Figure 1I,J). Moreover, the viral titers were markedly decreased at 6, 12, 24, 36 and 48 hpi in cells treated with ipriflavone (Figure 1K). Together, these results indicated that ipriflavone exhibited an inhibitory effect on PRRSV replication.

### 3.2. Ipriflavone Disturbs PRRSV Replication at Different Treatment Stages

**Figure 2:**
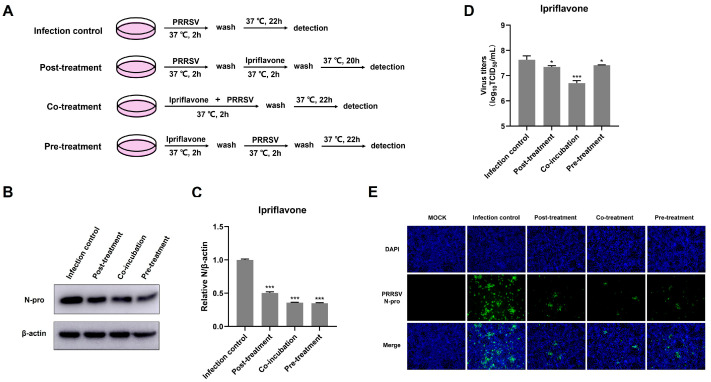
Ipriflavone suppresses PRRSV replication in various treatment stages. (**A**) Schematic diagram of experimental setup of ipriflavone and PRRSV (MOI = 0.1) treatments in Marc-145 cells. (**B**) PRRSV N protein levels were detected by Western blot. (**C**) The relative expression levels of N protein were quantified using Image J software. (**D**) The virus titers were analyzed by TCID_50_. (**E**) Immunofluorescence analysis of PRRSV N protein; Scale bar = 100 μm. The asterisks in the figures indicate significant differences (* *p* < 0.05, *** *p* < 0.001).

Next, we further examined whether ipriflavone could inhibit PRRSV replication under different treatment conditions as designed in Figure 2A. The results showed that ipriflavone significantly reduced the expression of PRRSV N protein, and the viral titers were also lower than those in corresponding control cells at all treatment stages (Figure 2B–D). Similarly, the fluorescence of PRRSV N (green) was reduced in different ways (Figure 2E). In particular, the inhibitory effect on PRRSV following post-treatment with ipriflavone was slightly less than that observed with co- or pre-treatment with ipriflavone. These results suggested that ipriflavone disturbed PRRSV replication and might act in both direct and indirect manners.

**Figure 3:**
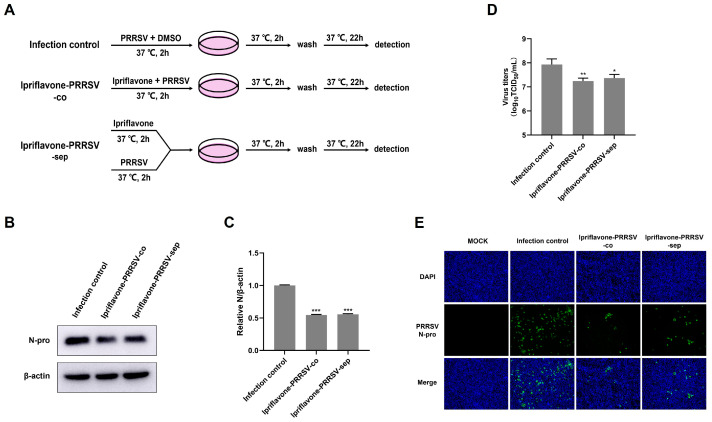
Ipriflavone could not directly reduce the infectivity of PRRSV. (**A**) Schematic of experimental setup of ipriflavone and PRRSV treatments in Marc-145 cells. (**B**) PRRSV N protein levels were detected by Western blot. (**C**) The relative expression levels of N protein were quantified using Image J software. (**D**) The virus titers were analyzed by TCID_50_. (**E**) Immunofluorescence analysis of PRRSV N protein; Scale bar = 100 μm. The asterisks in the figures indicate significant differences (* *p <* 0.05, ** *p <* 0.01, *** *p <* 0.001).

In order to investigate whether ipriflavone has a direct inhibitory effect on PRRSV, Marc-145 cells were treated with PRRSV-DMSO-co, PRRSV-ipriflavone-co, and PRRSV-ipriflavone-sep as shown in Figure 3A, and then the results were determined using Western Blot, TCID_50_ and IFA. The results indicated that co- or sep-incubation of ipriflavone and PRRSV significantly reduced the levels of N protein (Figure 3B,C) and virus titers (Figure 3D) in the virus-infected cells compared to the PRRSV-DMSO-co group, and the inhibitory effects had no significant difference between PRRSV-ipriflavone-co and PRRSV-ipriflavone-sep, and the similar results were visualized by the IFA assay (Figure 3E). Overall, these results suggest that ipriflavone did not directly reduce the infectivity of PRRSV.

### 3.3. Ipriflavone Inhibits PRRSV Infection During Virus Replication and Assembly Stage

**Figure 4:**
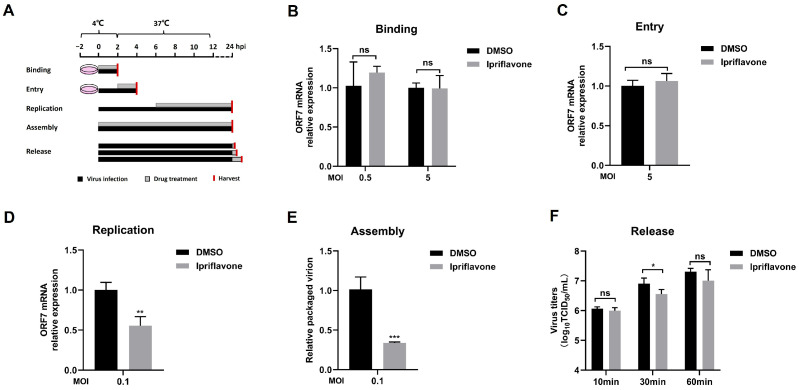
Ipriflavone blocks the replication and assembly of PRRSV. (**A**) Schematic of viral binding, entry, replication, assembly, and release assay. (**B**–**D**) The PRRSV ORF7 mRNA levels were determined by qPCR in the virus binding, entry, and replication assay. GAPDH was used as reference control. (**E**) The relative packaging efficiency of the viral genome was indicated by the ratio of viral titers to the copy number of the total viral genome. (**F**) In the virus release assay, virus titers of the cell supernatant were calculated by TCID_50_. The asterisks in the figures indicate significant differences (* *p <* 0.05, ** *p <* 0.01, *** *p <* 0.001, and ns, no significance).

Based on the inhibition of ipriflavone on PRRSV replication, we next designed binding, entry, replication, assembly, and release assays to further assess its inhibitory effect on viral life-cycle stages (Figure 4A). The results showed that no significant difference was observed in the binding and entry experiments when comparing the untreated group with the ipriflavone-treated groups (Figure 4B,C). Nevertheless, ipriflavone could affect PRRSV infection during virus replication (Figure 4D) and assembly stages (Figure 4E). Furthermore, ipriflavone affected the virus release stage after 30 min of treatment, but it failed to have any significant effect after 10 or 60 min of treatment. (Figure 4F). These results indicated that ipriflavone mainly blocked virus replication and assembly stages.

### 3.4. Ipriflavone Positively Regulates IFN-β Signaling

**Figure 5:**
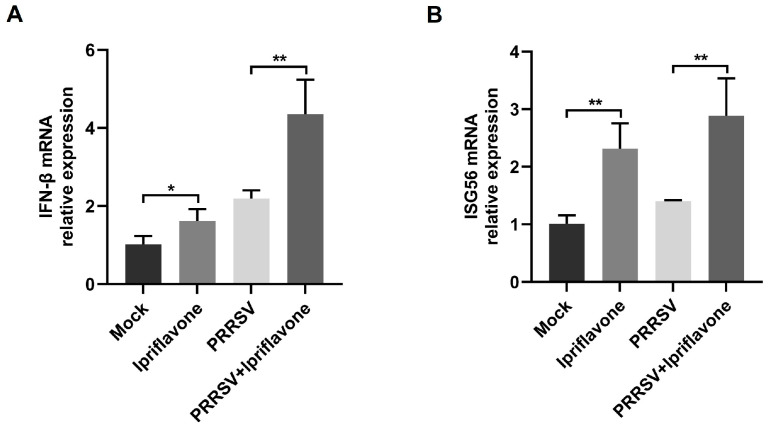
Ipriflavone effectively induced the activation of I-IFN signaling. Marc-145 cells were incubated with ipriflavone (20 μg/mL) and infected with or without PRRSV (MOI = 0.1) for 24 h, then the cells were collected. (**A**,**B**) The mRNA levels of IFN-β and ISG56 were measured by qPCR. The asterisks in the figures indicate significant differences (* *p <* 0.05, ** *p <* 0.01).

Type I-IFN signaling plays a crucial role in restricting virus infection [14,29]. Next, we wonder whether ipriflavone could regulate the production of type I-IFN. Compared to the control group, ipriflavone induced the transcription of IFN-β in the absence of virus and further enhanced the levels of IFN-β induced by PRRSV (Figure 5A). In addition, the transcription levels of ISG56 were also upregulated by ipriflavone regardless of whether there was PRRSV infection or not (Figure 5B).

### 3.5. Ipriflavone Inhibits PRRSV Replication by Promoting RIG-I/IRF3 Signaling Pathway

**Figure 6:**
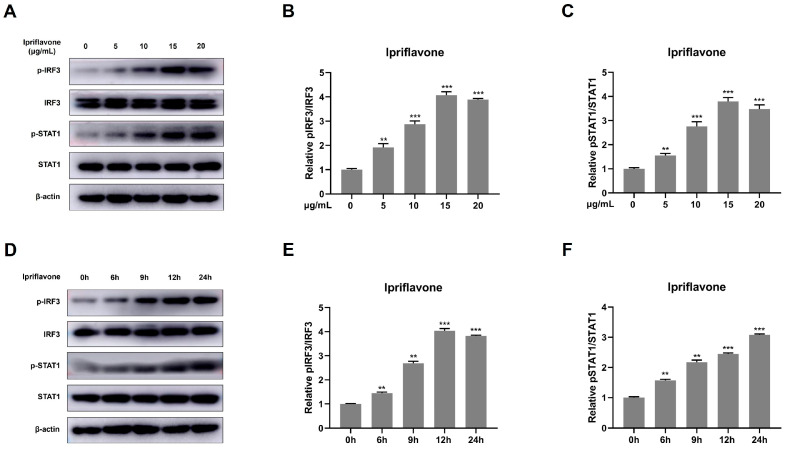
Ipriflavone effectively induced the activation of IRF3. Marc-145 cells were treated with ipriflavone (0, 5, 10, 15, 20 μg/mL) for 24 h. (**A**) The pIRF3, IRF3, pSTAT1, and STAT1 were detected by Western blot. β-actin was used as an internal control. (**B**,**C**) The relative expression levels of pIRF3 and pSTAT1 were quantified using Image J software. (**D**–**F**) The levels of pIRF3, IRF3, pSTAT1, and STAT1 were also detected in cells treated with ipriflavone at different times (0, 6, 9, 12, and 24 h) by Western blot. The asterisks in the figures indicate significant differences (** *p <* 0.01, *** *p <* 0.001).

RIG-I, the important component of RLR signaling, can recognize RNA viruses and then lead to the production of type I-IFNs and ISGs [30]. Herein, we investigated the phosphorylation of IRF3 and STAT1 in the presence of ipriflavone. The results showed that ipriflavone significantly induced the phosphorylation of IRF3 and STAT1 (Figure 6).

**Figure 7:**
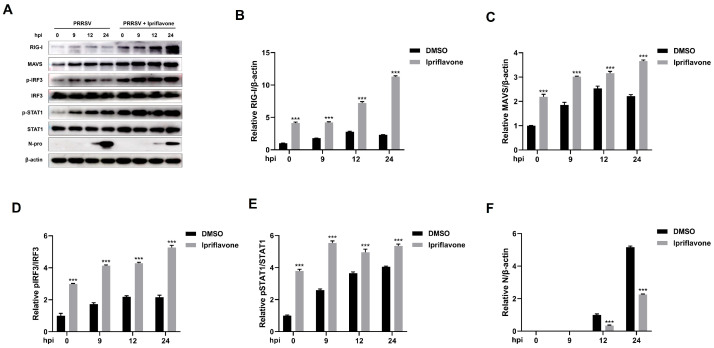
Ipriflavone inhibits PRRSV replication by promoting the activation of RIG-I/IRF3 signaling pathway. Marc-145 cells were mock treated or treated with ipriflavone and then infected with PRRSV (MOI = 0.1) for different time periods (0, 9, 12, and 24 hpi). (**A**) The RIG-I, MAVS, pIRF3, IRF3, pSTAT1, STAT1, and PRRSV N protein were detected by Western blot. (**B**–**F**) The relative expression levels of RIG-I, MAVS, pIRF3, pSTAT1, and N protein were quantified using Image J software. The asterisks in the figures indicate significant differences (*** *p* < 0.001).

**Figure 8:**
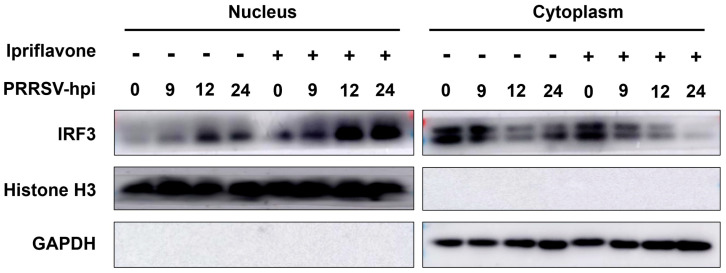
Ipriflavone treatment significantly promotes IRF3 nuclear translocation during PRRSV infection. Marc-145 cells were treated with ipriflavone in the absence or presence of PRRSV (MOI = 0.1). After 0, 9, 12, and 24 h, the cells were collected and the levels of IRF3 in the cytoplasmic and nuclear lysates were analyzed by Western blot with GAPDH and histone H3 as cytoplasmic fraction and nuclear fraction markers, respectively.

Subsequently, we investigated the effect of ipriflavone on RLR signaling upon PRRSV infection. As shown in Figure 7, ipriflavone treatment led to a significant upregulation in the expression levels of RIG-I and MAVS during PRRSV infection compared to control groups. Furthermore, it could increase the levels of pIRF3/IRF3 and pSTAT1/STAT1 at 9, 12, and 24 hpi, and the degree of phosphorylation exhibited a marked increase with increasing time. Meanwhile, the expression levels of PRRSV N protein were markedly lower in cells treated with ipriflavone. Additionally, we detected the nuclear translocation of IRF3 upon PRRSV and ipriflavone treatment. We found that ipriflavone significantly induced IRF3 nuclear translocation during PRRSV infection (Figure 8). Overall, these data demonstrated that ipriflavone could positively regulate the RIG-I/IRF3 signaling, which in turn is closely linked to the inhibition of PRRSV replication.

## 4. Discussion

PRRSV was initially discovered in both Europe and North America in the 1980s and has been widespread for over three decades in the global swine industry [31,32]. Vaccines against PRRSV have been used for many years in the pig industry. However, PRRSV remains one of the important economically damaging pathogens, suggesting that the existing vaccines may be unable to provide complete protection against PRRSV infection. Thus, novel strategies to control this disease are urgently needed. Natural compounds and their derivatives play important roles in antiviral response [33]. In this study, we demonstrated that ipriflavone exhibits anti-PRRSV activity.

Ipriflavone, a derivative of isoflavone, belongs to the flavonoids class and participates in the regulation of various physiological and pathological processes, including inflammation and apoptosis. However, the roles of ipriflavone in PRRSV infection are still unknown. In this study, we first explored the biological role of ipriflavone in PRRSV infection and found that ipriflavone exerted a significant inhibitory effect on PRRSV replication. Further investigation revealed that ipriflavone could disturb PRRSV replication at different treatment stages (post-, co- or pre-treatment with ipriflavone). And there was no significant difference in the inhibitory effects of ipriflavone between PRRSV-ipriflavone-co and PRRSV-ipriflavone-sep, suggesting that ipriflavone could not directly reduce the infectivity of PRRSV. In addition, ipriflavone could block virus replication and assembly but not the binding and entry stages. Bai et al. unveiled a novel functional role for full-length nsp2 of HP-PRRSV-2 in facilitating the assembly of the N protein with viral envelope proteins [34]. Additionally, it is reported that Galectin 3 binding protein (LGALS3BP) inhibits PRRSV replication by restricting both viral RNA synthesis and viral assembly [35]. EGCG restricts PRRSV proliferation, and analysis of the viral life cycle revealed that EGCG affected PRRSV replication and assembly, but not viral attachment, entry, or release [36]. Therefore, ipriflavone might block virus replication and assembly by inhibiting the synthesis of PRRSV-related protein or interacting with host proteins. Furthermore, ipriflavone could affect the virus release stage after 30 min of treatment, but it failed to exert any significant effect after 60 min of treatment. Based on these findings, we hypothesize that there might be some underlying mechanism that has led to this situation. Nevertheless, a more in-depth study is required in the future.

In addition, ipriflavone could enhance the expression of RIG-I and MAVS. As is well-known, innate immunity serves as the first effective line of host defense against pathogenic microorganisms [13]. RIG-I-like receptors are the important components of the innate immune response that can recognize and respond to RNA viruses [30,37]. When MAVS is engaged by RLRs, it can recruit downstream signaling complexes and lead to the activation of IRFs and NF-κB [38,39]. IRF3 activation is the hallmark of interferon pathway activation. In our study, we found that ipriflavone could promote the production of IFN-β and ISG56, and induce the phosphorylation and nuclear translocation of IRF3, which indicated that ipriflavone could positively regulate the RIG-I/IRF3 signaling, thereby inhibiting PRRSV replication. Myricetin and luteolin both belong to the category of flavonoids. It has been shown that myricetin could inhibit the proliferation of PRV by regulating the type I interferon signaling pathway [40]. Similarly, Wang et al. demonstrated that luteolin suppresses herpes simplex virus 1 (HSV-1) infection by activating the cGAS-STING pathway [41]. These findings collectively highlight the significant role of flavonoids in modulating antiviral immune responses. However, further in-depth research is needed to determine whether ipriflavone can also inhibit other PRRSV strains or viruses through the similar mechanism.

In addition, it has been shown that ipriflavone can attenuate the host inflammatory response related to NLRP3 inflammasome activation at implantation sites, and the anti-inflammatory role of ipriflavone in NLRP3 inflammasome activation is achieved through improving mitochondrial function [42]. In this study, ipriflavone induced both the phosphorylation and subsequent nuclear translocation of IRF3, thereby activating the downstream signaling. NF-κB is another key transcription factor in this cascade. Therefore, ipriflavone may also inhibit PRRSV replication by regulating NF-κB-mediated inflammatory response. Additionally, MAVS serves as a crucial innate immune adaptor protein located on the outer membrane of mitochondria [43]. Considering that ipriflavone could increase the expression level of MAVS, it is plausible that ipriflavone exerts its antiviral function via a certain mechanism by interacting with MAVS. However, further studies are required.

## 5. Conclusions

In summary, our study revealed the roles of ipriflavone in PRRSV infection and innate immune response. Here, we demonstrated that ipriflavone had the capacity to suppress PRRSV infection via inhibiting virus assembly stage and activating the RIG-I/IRF3 signaling pathway, which highlights the potential of ipriflavone as a therapeutic agent for inhibiting PRRSV replication and provides new insights into antiviral strategies.

## Figures and Tables

**Table 1:** Primers used for qPCR.

Primers	Forward Primer (5′ to 3′)	Reverse Primer (5′ to 3′)	GenBank No
ORF7	AAACCAGTCCAGAGGCAAGG	GCAAACTAAACTCCACAGTGTAA	AF331831
IFN-β	ACGGCTCTTTCCATGAGCTAC	GTCAATGCAGCGTCCTCCTT	NM001135795
ISG56	AGGAAACACCCACTTCGGTC	CCTCTAGGCTGCCCTTTTGT	XM015147649
GAPDH	GAAGGTGAAGGTCGGAGTCA	CATGTAAACCATGTAGTTGAGGTC	NM001195426

## Data Availability

All available data are presented in this manuscript.
